# A primary neuron culture system for functional studies of anoxia tolerance in turtles

**DOI:** 10.1242/jeb.250788

**Published:** 2025-12-18

**Authors:** Natalia A. Schneider, Claire L. Riggs, Mia Warmka, Seth Hofheins, Colton Smith, Daniel E. Warren

**Affiliations:** ^1^ Department of Biology, Saint Louis University, St Louis, MO 63103, USA; ^2^ Department of Biology, St Olaf College, Northfield, MN 55057, USA

**Keywords:** Neurophysiology, *Chrysemys picta bellii*, Vertebrates, Cortical neurons, Anoxia, Calcium recording, Fura-2, Glutamate

## Abstract

Cultured neuronal models for non-mammalian vertebrates are uncommon but could prove useful for investigating mechanisms of exceptional physiological performance, such as anoxia tolerance. We describe a procedure to isolate, culture and characterize cerebrocortical neurons of extremely anoxia-tolerant painted turtles; we then imposed anoxia while recording reactive oxygen species (ROS) using fluorescence photometry. Cerebrocortical sheets from hatchlings were dissociated enzymatically and mechanically, and cultured for ≤7 days. Within 24 h, most cells possessed classic neuronal morphology with identifiable soma and fine neurites. Immunocytochemistry showed MAP2-positive cells accounted for 90.9% of DAPI-positive cells. Ca^2+^ recordings (Fura-2) demonstrated neurons were immediately excitable with KCl and glutamate, but not acetylcholine. ROS recordings (CM-H2DCFDA) showed they avoided excessive ROS production post-anoxia, like other turtle brain preparations, but with higher signal and temporal resolution. These approaches should extend previous work in other brain preparations to isolated cells that possess the morphological and functional features of neurons.

## Teaser

**Summary:** Development of a novel model for neurophysiological studies of anoxia-tolerant vertebrates using isolated neurons from the cortex of the most anoxia-tolerant tetrapod – the western painted turtle.

## INTRODUCTION

Oxygen is essential for most animal life. Yet, some species, mostly ectotherms, have developed the physiological capacity to survive for extended periods in the complete absence of oxygen (i.e. anoxia). Unsurprisingly, most live in aquatic environments, where oxygen solubility is low and competition among aerobic organisms can be high. One champion of anoxia tolerance is the western painted turtle, *Chrysemys picta bellii*, adults of which have been repeatedly shown to survive for more than 170 days of anoxia at 3°C (Odegard et al., 2018; Ultsch and Jackson, 1982). Hatchling painted turtles can survive 40 days of anoxia at 3°C (Reese et al., 2004), and while this is less impressive than the adults, it is equal to the tolerance of adult red-eared slider, *Trachemys scripta elegans*, the other common model of anoxia tolerance in turtles (Warren et al., 2006). The mechanisms underlying this ability are multifaceted and often tissue specific, but almost universally rely on metabolic suppression.

Because of their high tissue-specific metabolic rates, and potential relevance to human health, much work has focused on excitable tissues, especially the brain (Buck and Pamenter, 2018; Hylland et al., 1997; Lutz, 1992; Lutz et al., 1996; Milton et al., 2007; Nilsson and Lutz, 2004). In mammals, approximately 60% of total brain ATP consumption is used by membrane Na^+^/K^+^-ATPase pumps to maintain ionic gradients and synaptic activity, while neurons expend roughly 80% of their total energy to generate excitatory activity (Pamenter et al., 2012; Raichle and Gusnard, 2002; Sokoloff, 1999). Thus, the ability to regulate energy consumption is critical for anoxia survival as anaerobic respiration yields less than 10% of the total ATP produced by aerobic respiration (Lutz and Nilsson, 1997).

The inability of most mammals to efficiently balance ATP consumption and production in response to oxygen deprivation leads to glutamatergic excitotoxicity and cell death resulting from ion-channel destabilization and extreme membrane depolarization (Bickler and Buck, 1998; Lipton, 1999; Pamenter et al., 2012). Over the last four decades, extensive work has significantly advanced our understanding of the neuroprotective mechanisms that allow anoxia-tolerant turtles to avoid these problems (for reviews, see Bickler and Buck, 1998; Buck and Pamenter, 2018; Lutz, 1992; Lutz and Nilsson, 2004; Lutz et al., 1996, 2003; Milton and Prentice, 2007; Nilsson and Lutz, 2004). Some of the hallmarks of anoxia tolerance include down-regulation of energy consumption and decreased ion channel activity and neuronal excitability. The critical balance between ATP demand and consumption has been well studied (Doll et al., 1994, 1991b; Hochachka, 1986; Hochachka et al., 1996; Hylland et al., 1997) where a reduction of ion channel conductance or ‘channel arrest’, particularly of K^+^ and Na^+^ channels, and NMDA and AMPA receptors (Buck and Bickler, 1998; Doll et al., 1991a; Pamenter et al., 2008a,b; Pék-Scott and Lutz, 1998; Pék and Lutz, 1997; Pérez-Pinzón et al., 1992b; Zivkovic and Buck, 2010) leads to a reduction in firing frequency or ‘synaptic arrest’ via an increase in GABA release (Buck and Pamenter, 2018; Lutz and Leone-Kabler, 1995; Pamenter et al., 2011; Pérez-Pinzón et al., 1992a) maintaining stable membrane potential through receptor downregulation (Bickler et al., 2000; Pamenter et al., 2008a), and decreased neuronal excitability and neurotransmission. More recently, Buck and colleagues have made significant contributions detailing the mechanisms of neuroprotection and the roles of mitochondria (for a review, see Hawrysh et al., 2022) in channel arrest via mitochondrial Ca^2+^ release (Dukoff et al., 2014; Pamenter et al., 2008b; Zivkovic and Buck, 2010), in spike arrest via decreased production of reactive oxygen species (ROS) (Hogg et al., 2015; Pamenter et al., 2007), and maintaining mitochondrial membrane potential via matrix acidification (Hawrysh and Buck, 2019a).

Despite these important advances in our understanding of neuronal anoxia tolerance, many gaps remain, particularly those related to survival mechanisms that are intrinsic to the neuron. This gap exists because most turtle studies of neuronal function during anoxia have utilized whole-tissue and multicellular preparations, especially the cortical sheet preparation first introduced by Blanton and colleagues (1989) and used extensively by Bickler, Buck and colleagues (e.g. Bickler, 1992; Buck and Bickler, 1998; Pamenter et al., 2012). Significant work has also utilized the isolated cerebellum (e.g. Perez-Pinzon et al., 1992a,b), or the intact brain of nitrogen-ventilated turtles (Chih et al., 1989; Milton et al., 2002; Pék-Scott and Lutz, 1998). The functional phenotype of the neurons recorded in these preparations will always reflect the recorded neuron's response not just to anoxia but also to the local mix of neurocrines, metabolites and small molecules produced from other cells in the tissue region during the anoxic imposition. Therefore, there is a need for a model to study the functional responses of isolated neurons to distinguish intrinsic responses resulting from anoxia from secondary responses to other stimuli present within the milieu.

Important early progress in the area was made by Milton and colleagues (2007), who developed a culture system of mixed, mitotic cell types derived from adult turtle brain. This pioneering work provided valuable insight into mechanisms by which turtle brain cells avoid injury from ROS (Milton et al., 2007; Nayak et al., 2009; Kesaraju et al., 2014; Reiterer and Milton, 2020). Although these cultures are NeuN (a marker of post-mitotic neurons) immuno-positive, they lack the full morphological and functional features of terminally differentiated neurons and do not respond robustly to depolarizing stimuli such as brevetoxin and glutamate (Cocilova and Milton, 2016).

Inspired by these foundational efforts, we have developed a complementary model to functionally characterize the intrinsic properties of isolated, primary neurons isolated from the cerebrocortex of hatchling painted turtles in response to an anoxic insult. In reptiles, the cerebrocortex is a three-layered structure (Naumann et al., 2015; Tosches, 2021), the middle of which is composed of a dense layer containing millions of pyramidal neurons (Laurent and Epstein, 2018; Shen and Kriegstein, 1986; Tosches et al., 2018). This is the same population of neurons studied by Buck and colleagues (Bickler et al., 2000; Buck and Pamenter, 2018; Lutz et al., 1996; Pamenter et al., 2008a) with the cortical sheet preparation, facilitating comparisons of function between isolated and multicellular preparations. We immunocytochemically, morphologically and functionally characterized these neurons using intracellular calcium recording techniques and then measured ROS production pre- and post-anoxia using fluorescence photometry at the single-neuron level.

## MATERIALS AND METHODS

### Animals

Western painted turtle, *Chrysemys picta bellii* (Gray 1831), eggs were collected annually in June 2020–2023 from multiple nest sites located in Rice Creek Chain of Lakes Park (Anoka County, MN, USA; MDNR Special Permits 29934-2020, 30386-2021/2022 and 35367-2023). Clutches were transferred to containers (11.4 cm width×21.6 cm length×3.8 cm height) with moistened grade-3 vermiculite (1.12 g sterile deionized water per gram of vermiculite, Uline Vermiculite, Pleasant Prairie, WI 53158, USA) and incubated at 25°C in a field incubator (Darwin Chambers, St Louis, MO, USA) for transport back to the laboratory. Eggs were then incubated in an environmental chamber (Darwin Chambers) at 25°C (to produce all males) and >95% humidity and checked daily until hatching in mid-August (8–9 weeks). Hatchlings were lightly brushed to remove any adhered vermiculite before weighing and then single-housed in black plastic cups without water (162 ml, 4.9 cm bottom diameter×7.6 cm top diameter×6 cm height). Temperature in the chamber ranged from 4 to 20°C from October until mid-April to simulate natural nesting conditions.

In early May, they were transferred to plastic tubs (68.5 cm width×45.7 cm length×31.7 cm height) containing 18–20°C dechlorinated, dealkalinized, St Louis City water. Water pH was adjusted to 8 using sulfuric acid or potassium hydroxide. The tubs were tilted approximately 6.5 deg to create a dry basking area, each equipped with one ZooMed^®^ 10 UVB 13 W compact fluorescent bulb (ZooMed^®^, San Luis Obispo, CA, USA) connected to timers to simulate a natural Minnesota photoperiod. For the first 2 weeks, hatchling turtles were fed frozen bloodworms, after which they were fed ZooMed^®^ Aquatic Turtle Food and dried black soldier fly larvae daily *ad libitum.* Water changes were carried out twice a week. All housing and animal procedures were approved by Saint Louis University (Protocols 2198 and 3151).

### Neuronal isolation

Primary neurons were isolated from the cortex of young western painted turtles (age 3–13 months; 2.57–8.96 g) and plated onto MilliCell EZ 4-well glass chamber slides (Sigma, St Louis, MO, USA) or Lab-Tek II CC2 4-well chamber slides (ThermoFisher Scientific, St Louis, MO, USA) for morphological and immunocytochemical analyses, respectively. We began the effort using CC2 slides, which are pretreated by the manufacturer with a proprietary coating and promoted as a simpler product to use. However, we switched to MilliCell EZ slides, which we coated with laminin and polyornithine (LPO) to be consistent with the plating procedure for the functional studies, which requires LPO coating (see details below). These consisted of 35 mm MatTek cover-slipped dishes (#1.5, MatTek, Ashland, MA, USA) for intracellular calcium recordings and 40 mm coverslips using 10×5 mm glass culture cylinders (Bioptechs, Butler, PA, USA) for the anoxia and ROS recording. All dissections and cell preparation were carried out on a clean bench (Purifier LABCONCO^®^, Kansas City, MO, USA) using aseptic technique and autoclaved instruments. All surfaces were disinfected either with UV or 70% ethanol. A summary of the workflow can be found in Fig. S1.

After placing on ice for 10–15 min, the turtles were quickly killed by decapitation. The head was sprayed with 70% ethanol and wiped with tissue, and the skull cap removed with scissors. The cerebral hemispheres (Fig. S2A) were cut from the olfactory bulbs and midbrain just rostral to the tectum, removed from the skull, and placed in ice-cold calcium and magnesium-free Hank's balanced solution (HBSS) with 5.5 mmol l^−1^ glucose (Gibco, Grand Island, NY, USA). Under a dissecting microscope with the tissue immersed, the cerebral hemispheres were separated, and the cortical sheet carefully dissected from the surrounding tissue, dorsal ventricular ridge, remaining dura matter and blood vessels using forceps and spring scissors (Fig. S2B). Using a plastic transfer pipette, the cortical sheets were briefly transferred to a new dish with fresh, ice-cold HBSS, bisected, then transferred to a 15 ml conical tube containing room temperature enzyme solution composed of Neurobasal medium without l-glutamine (Gibco) and the following additives: EDTA to 0.05 mmol l^−1^ (Corning, Manassas, VA, USA), CaCl_2_ to 1.5 mmol l^−1^ (Honeywell, Charlotte, NC, USA), l-cysteine hydrochloride monohydrate to 0.15 mmol l^−1^ (ThermoFisher Scientific) and papain to 1.54 mg l^−1^ (Worthington Biochem, Lakewood, NJ, USA). Prior to their addition to the Neurobasal, the CaCl_2_ and l-cysteine were stored at −20°C in aliquots of a single stock solution containing 100 mmol l^−1^ CaCl_2_ and 10 mmol l^−1^ l-cysteine. The papain enzyme solution in Neurobasal medium was pre-warmed to 37°C for 10 min, filtered, and held at room temperature for 30–60 min prior to use.

After the cortical tissue was added to the enzyme solution, the tube was placed in a 37°C water bath and lightly agitated every 5 min, just enough to keep the tissue from clumping. After 22 min, the tissue was rinsed twice using 2 ml of room temperature complete Neurobasal media (CNM), which is composed of Neurobasal without l-glutamine and with the following additives: fetal bovine serum to 4% (FBS; Atlas Biologicals; Fort Collins, CO, USA), l-glutamine to 2 mmol l^−1^ (Corning), Pen/Strep to 105 U ml^−1^ (Sigma) and B-27 to 2% (Gibco). The cortical tissue was transferred to 1.5–2 ml fresh, room temperature CNM for mechanical trituration by sequentially passing the tissue 1–3 times through three heat-polished glass pipettes with progressively smaller diameters ranging from 1.1 to 0.5 mm. Following mechanical trituration, 15–60 μl of suspended dissociated cells were transferred to each well of the MilliCell (see pre-treatment details below) and Lab-Tek CC2 chamber slides or MatTek dishes (see pre-treatment details below), immediately after which CNM was added. For each slide well, a small volume (∼50 µl) of CNM was added before the cells were transferred, followed by an additional 500 μl of CNM. For the MatTek dishes and 40 mm coverslips, 1.5 ml of CNM was added. Cells were then cultured at 30°C in an incubator with 4% CO_2_/balance HEPA-filtered room air for 1–7 days prior to use in morphological and immunocytochemical analyses or calcium recordings. This concentration of CO_2_ was selected because it produces a *P*_CO_2__ like those predicted to be found *in vivo* in 30°C-acclimated turtles.

### Dish preparation for isolated neurons

One day prior to their use, the MilliCell slides, MatTek dishes and 40 mm coverslips were coated with a solution composed of Ca^2+^- and Mg^2+^-free DPBS (Gibco) containing the following additives: laminin to 5 μg ml^−1^ (Sigma) and poly-l-ornithine to 50 μg ml^−1^ (Sigma). A total of 500 μl of coating solution was added to each chamber slide well, 200 μl to each Mattek dish and 300 μl to each coverslip and allowed to incubate for 2 h at room temperature. The solution was aspirated and the vessels rinsed 3 times with sterile PBS after coating for 2 h at room temperature. After aspiration, sterile PBS was added a fourth time and vessels were wrapped in Parafilm and stored at 4°C until their use the following day. Lab-Tek II CC2 chamber slides are precoated by the manufacture and so were not coated in the laboratory.

### Morphological analysis

For morphological analysis of primary cortical neurons, living cells were imaged at room temperature on days 1, 3, 5 and 7 under phase contrast on an Olympus CKX53 microscope using a 20× objective and photographed with an Olympus EP50 camera. After a quick survey for the presence of healthy cells, random images were taken of each chamber, averaging 16 images per slide. The image files were imported into ImageJ (v.1.54g) and the cells were counted and individually measured. Morphometric measurements included soma length with and without neurites, soma length of neuronal cells with one or more neurite, longest neurite length and number of neurites per cell. A total of 166 images and 3398 cells were measured from three young turtles for the morphological analyses.

### Immunocytochemistry

To immunolabel primary cortical neurons, cells in each chamber were first fixed with 0.5 ml of 4% paraformaldehyde (PFA; Sigma) in Ca^2+^- and Mg^2+^-free PBS (Corning) at room temperature on days 1, 3, 5 and 7 in culture. The solution was made fresh daily. The fixative was immediately removed to rinse away residual media, and then followed by a second addition of 4% PFA that was allowed to fix cells for 10 min. The wells were rinsed 3 times with PBS, followed by the addition of 1 ml of 0.02% sodium azide/PBS solution to each well to prevent biofilm formation during storage. Slides were wrapped in Parafilm and stored at 4°C for up to 2 weeks prior to permeabilization and immunolabeling studies.

Cells were permeabilized with 1 ml of 0.3% Triton™ X-100 (ThermoFisher Scientific) in Ca^2+^- and Mg^2+^-free PBS for 5 min at room temperature and rinsed 3 times with PBS (5 min each). Cells were incubated for 1 h at room temperature in 500 µl of blocking solution consisting of 5% donkey serum (Southern Biotech, Birmingham, AL, USA) in PBS. After 1 h, the blocking solution was replaced with 400 µl of blocking solution containing the primary antibodies and incubated at 4°C. The primary antibodies used included mouse anti-glial fibrillary acidic protein (GFAP) monoclonal antibody (1:500 or 1:250; Invitrogen, Waltham, MA, USA, ref: 14-9892-82, lot 2297222), and rabbit anti-microtubule associated protein-2 (MAP2) polyclonal antibody (1:500; Abcam, Waltham, MA, USA, ref: ab32454, lot gr3324658-1). Similar results were obtained with both dilutions of GFAP, so the data were ultimately pooled for analysis. After 24 h, primary antibodies were removed, and the cells rinsed 3 times with PBS (5 min each). For secondary immunolabeling, cells were incubated for 1 h in the dark, at room temperature, in 400 µl of blocking solution containing donkey anti-rabbit IgG Alexa Fluor 488 and donkey anti-mouse IgG Alexa Fluor 594 (both 1:500; Invitrogen). Cells were then rinsed 3 times with PBS (5 min each), and once with molecular grade water (Corning) for 5 min. Finally, cells were stained with 1 mg ml^−1^ DAPI (Biotium, Fremont, CA, USA) in PBS for 1 min in the dark, at room temperature, rinsed 3 times with PBS (5 min each), cover-slipped with Aqua-Poly/Mount (Polysciences, Warrington, PA, USA), dried overnight in the dark at room temperature, and stored at 4°C prior to imaging. To assess the potential for non-specific binding of primary polyclonal antibody, cells in one chamber of each slide were always incubated with only the 5% blocking solution, secondary antibody and DAPI.

Immunofluorescent cells were imaged with a 10× objective on a Leica SP8 confocal microscope (NSF MRI grant number 1253939), excited with 405, 488 and 522 nm diode lasers and collected via two HyD and two PMT detectors. Cell data were acquired and analyzed using Leica Application Suite X (LAS X). A total of 10 random squares were imaged from each chamber using the randomized image acquisition tool. A maximum projection was created for each of the 10 images. Cells were automatically counted using the automated cell counting tool. A cell count template was created with pre-set lower and upper threshold intensities bound for each parameter: MAP2+DAPI positive cells were >80% MAP2 positive and <50% GFAP; GFAP+DAPI positive cells were >80% GFAP positive and <50% MAP2; MAP2+GFAP+DAPI positive cells were >80% MAP2 and >80% GFAP positive.

### Intracellular calcium ([Ca^2+^]_i_) recording

Cells were loaded at room temperature with 5 μmol l^−1^ Fura-2 AM (Invitrogen) for 30 min in extracellular solution containing: HBSS augmented with 10 mmol l^−1^ Hepes (Sigma), 2.5 mmol l^−1^ CaCl_2_ (Honeywell) and 2 mmol l^−1^ MgCl_2_ (ThermoFisher Scientific). The pH was adjusted to 7.4 with 1 mol l^−1^ NaOH (ThermoFisher Scientific). After loading, cells were washed with extracellular solution and allowed to sit at room temperature for 15 min before a perfusion insert (PCP-1, AutoMate Scientific, Philadelphia, PA, USA) was placed in the dish, which was then mounted to the QuickStage™ microscope dish holder (QS-U-35, AutoMate Scientific). The cells were perfused with extracellular solution through the ports of the PCP-1 chamber using an eight-channel manifold (MAN81; Cell MicroControls, Norfolk, VA, USA).

Δ[Ca^2+^]_i_ was recorded with a PMT (D-104 PTI, Horiba, Piscataway, NJ, USA) and monochromator (DeltaRAM X, Horiba) based fluorescence photometry system on an Olympus IX51 microscope using a high NA 40× oil objective. The monochromator and PMT were interfaced to a computer with an A/D converter (ASOC-10, Horiba), all of which were controlled by FeliX 4.9 software (Horiba). Individual cells were excited intermittently at 340 or 380 nm while counting the photons emitted at 510 nm. Background emissions were subtracted from each recording before being expressed as the ratio (*R*) of 340/380 nm fluorescence emission. Data were exported and analyzed using ClampFit 10.7 (Molecular Devices, San Jose, CA, USA). Fluorescence emission was recorded while the cells were perfusion-pulsed, for 10–15 s, either with extracellular solution containing 20 mmol l^−1^ KCl, 100 μmol l^−1^ glutamate or 100 μmol l^−1^ acetylcholine. Cells were perfused with extracellular solution before and after the perfusion pulses until 340/380 ratios returned to baseline levels (<1 min). Perfusion and flow rates were controlled by a cFlow 8 Ch and cF-8VS valve assembly (Cell MicroControls) with typical flow rate of 1.4 ml min^−1^.

### ROS measurements

Cells plated to 40 mm coverslips were loaded at 30°C with 5 µmol l^−1^ of the dye 6-chloromethyl-2′,7′-dichlorodihydrofluorescein diacetate, acetyl ester (CM-H_2_DCFDA; Molecular Probes, Eugene, OR, USA) for 10 min in turtle artificial cerebral spinal fluid (aCSF) containing (in mmol l^−1^): 107 NaCl, 2.6 KCl, 1.2 CaCl_2_ (Honeywell), 1.0 MgCl_2_, 2.0 NaH_2_PO_4_, 26.5 NaHCO_3_, 10.0 glucose (Sigma) and 5.0 imidazole (Pfaltz & Bauer, Waterbury, CT, USA) to diH_2_O (Lari and Buck, 2021). Turtle aCSF pH was titrated to 7.6 with 1 mol l^−1^ NaOH. After incubation, cells were allowed to reach room temperature and washed with turtle aCSF for 5 min.

After washing, 40 mm coverslips were quickly mounted to a FCS2 laminar flow perfusion chamber (Bioptechs) and room temperature turtle aCSF was quickly added to the coverslip to prevent cells from desiccating. The cells were perfused through the port of the FCS2 chamber using an eight-channel manifold (MAN81, Cell MicroControls). For experiments with normal O_2_ conditions (i.e. normoxia), solutions were bubbled with 97% air/3% CO_2_ and perfused through individual perfusion lines containing normoxic aCSF and normoxic pyrogallol (50 µmol l^−1^; Fluka Analytical, Charlotte, NC, USA). For experiments without oxygen (i.e. anoxia), solutions were bubbled with 97% N_2_/3% CO_2_ and perfused through an individual Viton perfusion line containing anoxic aCSF. Oxygen levels were measured using a FireSting GO2 oxygen meter recording fluorescence from a sensor spot mounted on the inside of the top glass of the perfusion chamber (OXKIT-CTL, Pyroscience, Aachen, Germany).

Δ[ROS]_i_ was recorded with the same equipment and software as described for Δ[Ca^2+^]_i._ Background emissions were subtracted from each recording before being expressed as change in emission intensity across time. CM-H_2_DCFDA was excited at 488 nm and emitted photons were detected at 540 nm (ET 540/40m, 25 mm). Data were exported and analyzed using ClampFit 11.3 (Molecular Devices).

### Statistical analysis

Statistical analysis was carried out in R Studio (v.2024.09.1+394 ‘Cranberry Hibiscus’). Normality and homogeneity of variances were calculated for each dataset using Shapiro–Wilk test and Bartlett's test, respectively, before running a one-way ANOVA or two-way ANOVA with interaction test. If assumptions were violated, data were root-square transformed or ranked prior to the analysis. A significant outcome was followed by a *post hoc* Student–Newman Keuls (SNK) test to identify the differences among groups. If assumptions were violated after data transformations, a one–way ANOVA Kruskal–Wallis test was used. A significant outcome was followed by a Conover–Iman test which is robust to unequal variances. Differences were significant when *P*<0.05.

## RESULTS AND DISCUSSION

The goal of this work was to devise an *in vitro* system for characterizing the functional responses of isolated cortical neurons from the most anoxia-tolerant tetrapod, the western painted turtle. This system utilizes primary neurons isolated from the cerebrocortex of hatchling turtles that (1) exhibit the expected morphological characteristics of cortical neurons, including rounded soma and thin neurites and (2) show positive MAP2 immunoreactivity; (3) cells meeting our morphological criteria exhibited a stereotypical neuronal functional phenotype, i.e. immediate sensitivity to the excitatory neurotransmitter glutamate; (4) these bona fide neurons showed a predictable response to an anoxic insult; specifically, reductions in intracellular ROS levels, only a slight increase during the reperfusion period and suppression during recovery.

### Morphology studies

**Fig. 1.**
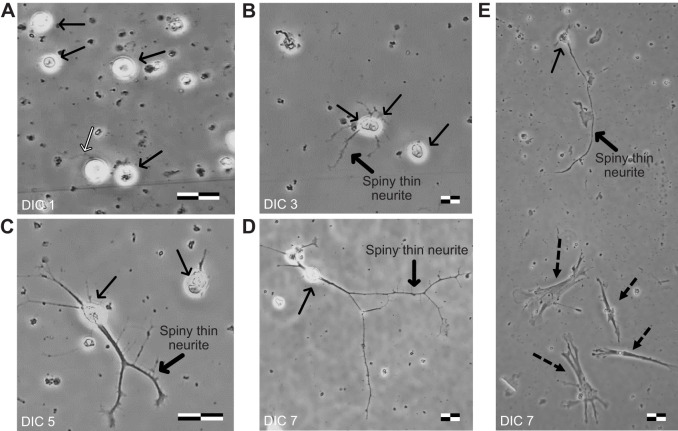
**Representative images of isolated primary cortical neurons from *Chrysemys picta bellii* turtles during the first week in culture.** Images and morphological measurements were made across 7 days in culture (DIC) on days 1, 3, 5 and 7. (A) On DIC 1, cells were bright, with round and teardrop-shaped soma indicated by black arrows. The neurite is indicated by the white arrow. Scale bar: 15.5 µm. (B) On DIC 3, cells had teardrop-shaped soma and started to show the growth of thin neurites. A spiny, thin neurite is indicated by the thick black arrow. Scale bar: 21 µm. (C) On DIC 5, pyramidal neurons showed developing basal and apical branched neurites. Scale bar: 22 µm. (D) On DIC 7, pyramidal neurons such as this one showed highly developed branched neurites. Scale bar: 13 µm. (E) A side-by-side comparison of neurons and fibroblasts on day 7. The pyramidal neuron shows highly developed branched neurites. The fibroblasts are indicated by dashed arrows. Scale bar: 13 µm.

For a cultured cell to be useful for any neurophysiological studies, it must exhibit the cellular morphology normally associated with neurons. We characterized the morphology of cells prepared for the cerebrocortices of three animals using wide-field microscopy and found that neurons developed across 7 days in culture (DIC) and exhibited the morphological features of cortical neurons (Fig. 1A–D). Morphological measurements are summarized in Table S1. On day zero, cells were numerous, with rounded or tear-shaped soma (Fig. S3). A few cells retained neurites and others formed sparse, small clusters of incompletely dissociated neurons while suspended in the growth medium (Fig. S3, checkered arrows). On DIC 1 (Fig. 1A), cells were bright with rounded or tear-shaped soma (mean±s.e.m. length 12.62±0.15 µm, *n=*828 cells) and most were adhered to the glass apart from smaller cells that were only partially adhered. Some cells had singular or multiple thin neurites (2.47±0.24 neurites per cell, *n=*94 cells with neurite), with a mean±s.e.m. length of the main neurite of 35.23±2.42 µm; however, most cells lost neurites as a result of the dissociation process. On DIC 3 (Fig. 1B), cells were well adhered, with more cells showing tear-shaped soma (length 13.84±0.16 µm, *n*=829), and starting to show regeneration of neurites (2.70±0.15 neurites per neuron, length 53.65±4.45 µm, *n*=239 cells with neurites). Although many cells remained without or with short neurites, some showed long and thin neurites, and others had long, spiny and branched ones. Synapses could already be observed forming in densely seeded areas of the slide (Fig. S4B,F, striped arrows). On DIC 5 (Fig. 1C), most cells displayed neurites (4.10±0.22 neurites per neuron, 74.78±5.09 µm long, *n*=313 cells with neurites); many had well-developed spiny, thin and highly branched neurites. Most cells had tear-shaped soma (11.71±0.18 µm length, *n*=782). Distinct morphologies could be observed beyond multipolar cells, including unipolar and bipolar. On DIC 7 (Fig. 1D), cells showed larger soma (length 16.52±0.20 µm, *n*=859 cells) with numerous, highly branched neurites (4.72±0.22 neurites per neuron, length 114.79±5.34 µm, *n*=430 cells with neurites). Densely seeded areas of the slide contained multiple synapsing neurons, whereas sparsely seeded areas contained numerous isolated neurons. Fibroblasts, when present, were frequently observed in confluence (Fig. S5B). However, when observed in isolation, they were easily distinguishable from neuronal cells, as fibroblasts were flat, spindle-shaped elongated cells with a large nucleus, and found in mitotic clusters (Fig. 1E and Fig. S5, dashed arrows). The neurons in the cultures could not be described as confluent. Contact points between neurons and fibroblasts were also observed (Fig. S5A,C, gray arrows). The results comparing the two types of chamber slides are shown in Fig. S4 and Table S2. Both slides yielded cells that were well attached, but laminin/polyornithine-coated slides yielded more neurons with well-developed neurites than factory-ready, untreated CC2 slides.

### Immunolabeling studies

**Fig. 2.**
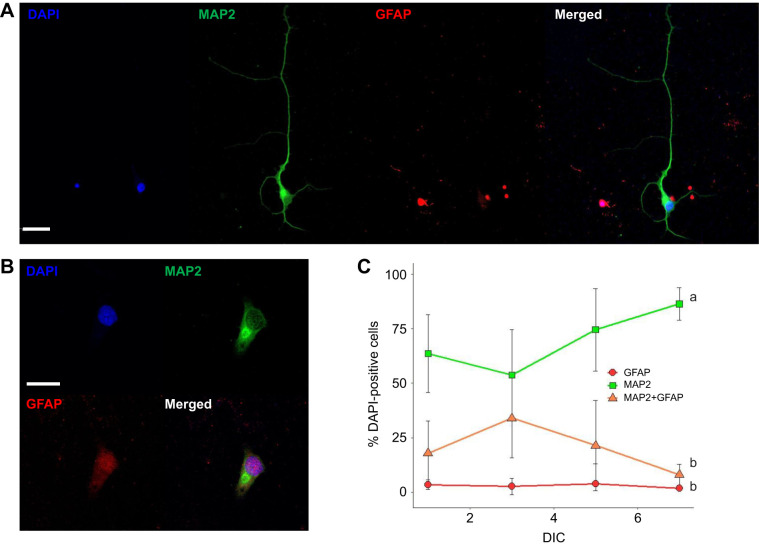
**Immunofluorescence images of primary neurons isolated from neuronally rich regions of the *C. picta bellii* cortex.** (A) Cells were fixed with 4% paraformaldehyde (PFA) on DIC 3 and labeled with DAPI (nuclei), Alexa Fluor 488 (MAP2 neuronal marker) and Alexa Fluor 594 (GFAP glial marker). Images were acquired using a 40× oil immersion lens on a Leica SP8 confocal microscope. Scale bar: 25 µm. (B) Immunolabeling of a fibroblast on DIC 3, showing expression of both MAP2 and GFAP. Scale bar: 20 µm. (C) Mean percentage of DAPI-positive cells that were immunolabeled on DIC 1, 3, 5 and 7. MAP2-positive cells (neurons) accounted for most cells in all samples (90.9%, green squares), whereas GFAP-positive cells accounted for just 2.3% (red circles) and the remaining 7.04% of cells (orange triangles) were positive for MAP2+GFAP. Differing lowercase letters indicate statistical differences among immunolabeled groups (two-way ANOVA, *F*=41.003, *P*<0.001), no statistical difference among DIC (*P*=0.918), and no effect of interactions between immunolabeling and time in culture (*P*= 0.561). Data are means±s.e.m., *n*=3–5 animals per group.

It is common practice to identify cell types in cultures using immunocytochemistry. Using primary antibodies with affinities for MAP2 (microtubule-associated protein-2), a cytoskeletal protein found in neurons, and GFAP (glial fibrillary acidic protein), a cytoskeletal protein found in glia, we found that 90% (*n=*13,698 cells from five turtles) of DAPI-positive cells (>97% of the fixed cells) expressed MAP2 (Fig. 2A), further indicating that the cultures were neuronally enriched. This number includes those that were immuno-positive for both MAP2 and GFAP (Fig. 2B), which accounted for 7.04% of the cells counted. We attribute the double-labeling to the presence of a small number of non-neuronal cells, as some have been shown to express both GFAP and MAP2, including fibroblasts (Bloom and Vallee, 1983; Hainfellner et al., 2001) and astroglial precursor cells (Rosser et al., 1997). Just 2.5% of DAPI-positive cells were immunolabeled for GFAP, which is found in glial cells such as astrocytes and ependymal cells. The percentage of immunolabeled cells for each antibody did not vary across DIC and the number of MAP2 immunolabeled cells was always greater than GFAP only and GFAP/MAP2 double-labeled cells (two-way ANOVA, *F*=41.003, *P*<0.001, SNK *post hoc*, *P*<0.001) (Fig. 2C).

### Functional characterization

**Fig. 3.**
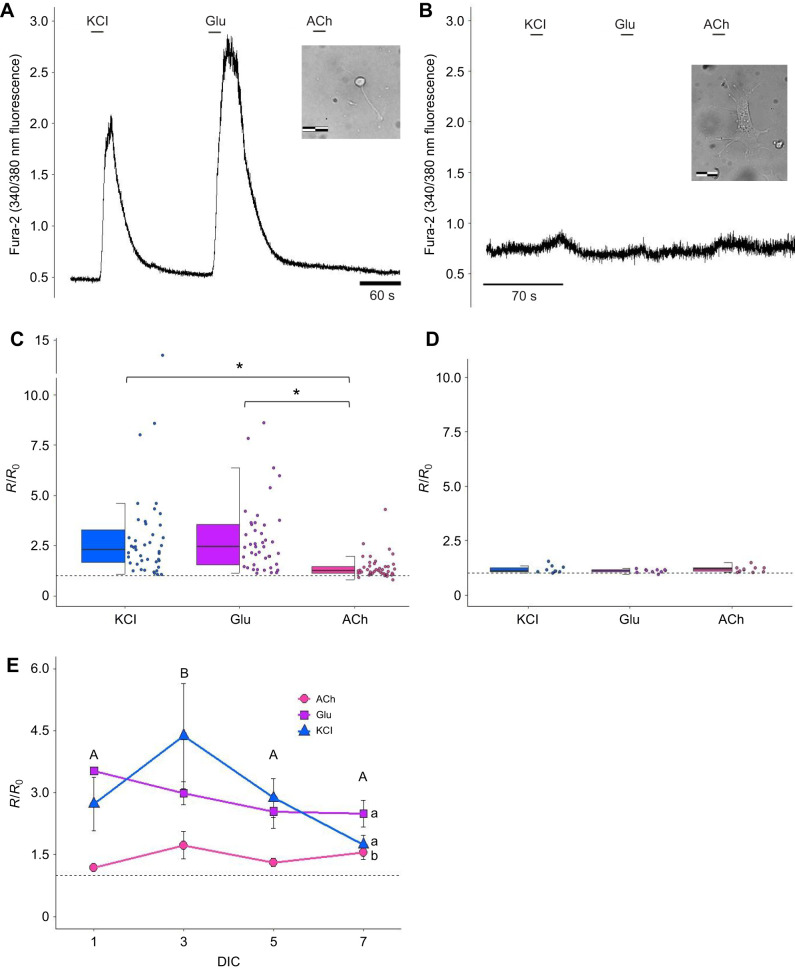
**Calcium recording of isolated primary neurons and fibroblasts from *C. picta bellii*.** Primary neurons were excitable (20 mmol l^−1^ KCl) and expressed glutamate receptors (100 µmol l^−1^ Glu), showing only slight responses to acetylcholine (100 µmol l^−1^ ACh). Fibroblasts showed no functional responses to the treatments. (A) Example trace of calcium recording (ratio *R* of Fura-2 340/380 nm fluorescence emission) of one isolated primary neuron at DIC 1 (inset; scale bar: 22 µm). (B) Example trace of calcium recording of one isolated fibroblast DIC 5 (inset; scale bar: 22 µm). (C) Change in fluorescence (*R*/*R*_0_) of a total of 43 cells (neurons) recorded from 9 turtles (DIC 1–7). Box plots show median, upper and lower quartiles and 1.5× interquartile range; individual data points are shown to the right. A two-way ANOVA on ranks showed a significant difference between groups (*P*<0.001). A Student–Newman–Keuls (SNK) *post hoc* test revealed that the response to ACh was significantly different from that to KCl (*P*<0.001) and Glu (*P*<0.001). Statistical significance is denoted by asterisks (*P<0.001). (D) A total of 10 fibroblasts were recorded from 4 turtles (DIC 5–26). A one-way ANOVA on ranks showed no statistically significant difference between groups (*P*=0.49). (E) Mean±s.e.m. *R*/*R*_0_ ratio for a total of 43 neurons across DIC (*n*=9–15). There were statistically significant differences among treatments (two-way ANOVA, *F*=30.160, *P*<0.001) and across DIC (two-way ANOVA, *F*=3.895, *P*=0.0108) but no interaction between DIC and treatment (*P*=0.3988). Significant differences between groups with *P*<0.05 are denoted by differing lowercase letters across treatments, and by differing uppercase letters for comparisons across DIC. Dashed line represents *R*/*R*_0_=1.0.

Although the morphology and immunolabeling techniques may indicate that a cell has a neuronal appearance, the most important property for identifying a neuron is that it is excitable and immediately responsive to depolarizing stimuli and excitatory neurotransmitters. We measured neuronal excitability using Fura-2 fluorescence photometry while perfusing Fura-2-loaded cells (5 µmol l^−1^; *n*=43, 9 turtles) with the excitatory agents KCl (20 mmol l^−1^), glutamate (100 µmol l^−1^) and acetylcholine (100 µmol l^−1^). Ten second applications of KCl and glutamate produced immediate and robust Ca^2+^ transients that were similar in magnitude to one another (Fig. 3A; two-way ANOVA, *F*=30.160, *P*<0.001, SNK *post hoc*; *P*>0.05). Responses to acetylcholine application were significantly smaller (*P*<0.001; Fig. 3C). From this, we conclude that the majority of these primary neurons are excitable, and possess glutamate receptors and voltage-gated calcium channels, but few cholinergic receptors. This result was validating, as the cortical sheet is not known to be rich in cholinergic neurons (Powers and Reiner, 1993).

Glutamate sensitivity was tested over a 7 day period, and we found no interactions between DIC and excitability or sensitivity to glutamate (Fig. 3E; two-way ANOVA, *F*=1.047, *P*=0.3988). The comparison among the DIC time points, where treatment responses were grouped by day in culture, showed DIC 3 cells were slightly, but significantly, more responsive (two-way ANOVA, *F*=3.895, *P*=0.0108) than cells at all other days in culture. No statistical differences were found among the other DIC time points, indicating that we detected similar functional responses across the first week in culture.

We also made the same measurements on cells with a clear fibroblast morphology (*n*=10 cells, 4 turtles). Predictably, we observed no calcium transient after a 10 s application of any of the three treatments (Fig. 3B) and there was no significant variation among the groups (Fig. 3D; one-way ANOVA, *F*=0.733, *P*=0.49).

### ROS production during anoxia

**Fig. 4.**
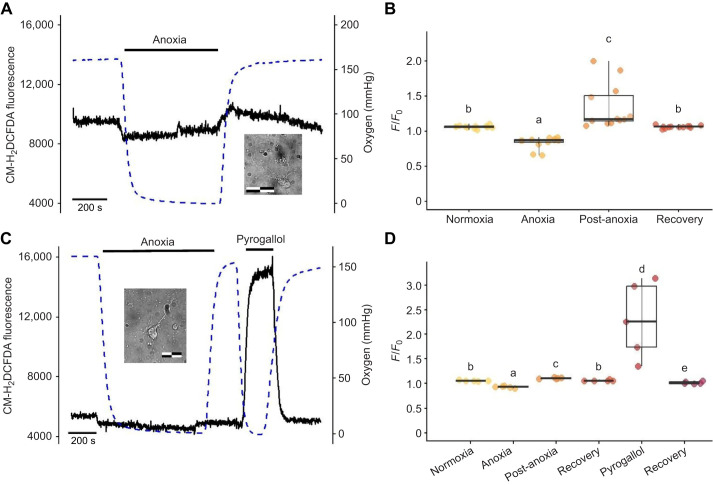
**Reactive oxygen species (ROS) recordings in isolated primary neurons from *C. picta bellii*.** Primary neurons were cultured for 3–7 days and loaded with ROS indicator CM-H_2_DCFDA (488 nm fluorescence emission). They showed a small spike in ROS production during the initial post-anoxia reoxygenation period (∼70 s) but not during the transition from normoxia to anoxia (97% N+3% CO_2_), and a large spike with perfusion of 50 µmol l^−1^ of the ROS generator pyrogallol. (A) Representative recording of CM-H_2_DCFDA fluorescence in an isolated primary neuron during normoxia, anoxia and recovery (inset; scale bar: 40 µm). Oxygen levels are shown on the right *y*-axis (dashed line). (B) A total of 12 cells were recorded from three turtles. CM-H_2_DCFDA fluorescence signal (*F*/*F*_0_, 488 nm) decreased (*P*<0.001) during anoxia, indicating a decrease in production of hydrogen peroxide (H_2_O_2_) and superoxide (O_2_·^−^). Upon initial post-anoxia reoxygenation CM-H_2_DCFDA fluorescence increased (*P*<0.001), indicating ROS production levels higher than normoxic controls. During recovery, CM-H_2_DCFDA fluorescence returned to normoxic control levels (*P*>0.05). Box plots show median, upper and lower quartiles and 1.5× interquartile range. (C) Representative recording of CM-H_2_DCFDA fluorescence in an isolated primary neuron during normoxia, anoxia, recovery, perfusion of 50 µmol l^−1^ of the ROS generator pyrogallol, and recovery (inset; scale bar: 40 µm). Perfusion with pyrogallol resulted in a concomitant decrease in oxygen (dashed line) to anoxic levels. (D) A total of 5 cells were recorded from 2 turtles. ROS production decreased (*P*<0.001) during anoxia but increased above normoxic controls upon initial post-anoxia reoxygenation (*P*<0.001). Perfusion of 50 µmol l^−1^ of pyrogallol resulted in increased ROS production when compared with normoxic controls (*P*<0.001) and with post-anoxia reoxygenation (*P*<0.05). Reperfusion with oxygenated solution decreased CM-H_2_DCFDA fluorescence to levels below normoxic controls (*P*<0.01). Data were analyzed by one-way ANOVA Kruskal–Wallis followed by a Conover–Iman *post hoc* test (*P*<0.05). Normoxic and recovery solution were bubbled with 97% air+3% CO_2_.

To further demonstrate the utility of the system for studies of anoxia, we measured general ROS production in neurons loaded with CM-H_2_DCFDA using fluorescence photometry before, during and after perfusion with anoxic turtle aCSF, as well as the ROS generator pyrogallol. In both experiments, we detected significant and dynamic changes in ROS production (Fig. 4B; one-way ANOVA Kruskal–Wallis, χ^2^=38.96, *P*<0.001; Conover–Iman *post hoc* tests). During anoxia, the cortical neurons (*n*=12 cells, 3 turtles) showed suppression of net ROS production during 10 min of anoxic perfusion (Fig. 4A; *P*<0.001). During post-anoxia reperfusion, ROS levels increased slightly above the normoxia baseline during the first ∼70 s of the reoxygenation period (*P*<0.001), followed by a return to the normoxia baseline within 5–9 min after the reoxygenation peak (*P*>0.05). We observed decreases below the normoxia baseline in five cells, but the differences were not statistically significant. The overshoot during recovery (Fig. 4A) could result either from mechanisms that suppress ROS levels or from a delay in the restoration of oxidative phosphorylation rates during the initial reperfusion period.

In a subset of experiments, ROS production was stimulated as a positive control during normoxia with 50 µmol l^−1^ of pyrogallol (*n*=5 cells, 2 turtles), producing dynamic changes in ROS and oxygen levels (Fig. 4C; one-way ANOVA Kruskal–Wallis, χ^2^=26.73, *P*<0.001; Conover–Iman *post hoc* test). Cellular ROS levels increased (*P*<0.001) with pyrogallol while oxygen was depleted within the perfusion chamber. Upon reperfusion with normal aCSF, ROS fell below normoxic controls (*P*<0.001; Fig. 4D) to levels observed during the anoxia perfusion, suggesting activation of processes to decrease cellular ROS levels.

### Significance and impact

The present work is the first to describe a procedure for isolating, culturing and investigating the physiological responses of cerebrocortical neurons from an anoxia-tolerant species, where the cells themselves have been morphologically and functionally characterized. The neurons showed immediate, robust excitability to KCl and glutamate and displayed ROS suppression during anoxia and post-anoxia reoxygenation. The morphological and physiological features of turtle cortical neurons have previously been characterized in cortical slices (Blanton and Kriegstein, 1991; Larkum et al., 2008; Shen and Kriegstein, 1986; Tosches, 2021) and functional measurements of anoxia tolerance have been extensively examined in turtle cortical sheets and other brain tissue preparations (Bickler et al., 2000; Buck and Pamenter, 2018; Lutz et al., 1996; Pamenter et al., 2008a). Confluent, mixed cell cultures from whole-brain regions have also been used to elucidate the mechanisms of ROS suppression (Kesaraju et al., 2014; Milton et al., 2007; Nayak et al., 2009; Reiterer and Milton, 2020). However, we are unaware of any studies that successfully cultured primary cortical neurons from turtle brain that exhibit typical neurophysiological activity (i.e. excitability, immediate glutamate sensitivity, etc.). The main reason for this gap has been the difficulty in isolating healthy primary neurons from adult tissue, which is not unique to reptiles (Brewer, 1997; Brewer and Torricelli, 2007). A widespread practice among mammalian neuroscientists is to culture neurons from neonatal and embryonic rodents because cell viability and survival are increased (Belle et al., 2018). A key innovation was to extend this strategy to an anoxia-tolerant species by isolating and culturing neurons from the cerebrocortex of yearling painted turtles.

Our results demonstrated that isolated cerebrocortical neurons possess intrinsic mechanisms that suppress ROS production during anoxia, and allow for only a small, though statistically significant accumulation of ROS during initial post-anoxia reoxygenation. This real-time profile of intracellular ROS during anoxia was similar to measurements previously made in cortical sheet preparations by Buck and colleagues (Dukoff et al., 2014; Hogg et al., 2015; Pamenter et al., 2007). However, our work demonstrates several key advantages, including greater signal and temporal resolution, and the ability to achieve a relatively stable baseline fluorescence level and quantify rapid changes in ROS levels (within seconds) that can be directly correlated to chamber *P*_O_2__ as oxygen is flushed from and reintroduced into the chamber (Dukoff et al., 2014; Hawrysh and Buck, 2019b; Hogg et al., 2015; Pamenter et al., 2007).

The work is consistent with the ROS suppression observed by Milton and colleagues in mixed, confluent cell cultures prepared from adult turtle brain, which showed that adenosine, neuroglobin, upregulation of Hsp72 and induction of foxo3a improve cell survivability by suppressing ROS accumulation after 1–4 h of anoxia (Kesaraju et al., 2014; Milton et al., 2007; Nayak et al., 2009; Reiterer and Milton, 2020). In addition to these potential mechanisms, future work with isolated neurons should examine responses to longer anoxic periods and interrogate the importance of the many unstudied extrinsic modifiers, such as pH, lactate and other small molecules known to change during anoxia. Taken together, this work demonstrates the potential of this *in vitro* model to greatly enhance our understanding of the intrinsic and extrinsic determinants of anoxia tolerance in vertebrate neurons.

### Conclusion

We have successfully established a protocol for isolating and culturing primary cortical neurons from the most anoxia-tolerant tetrapod, the western painted turtle. These neurons show characteristic morphology, expression of a neuronal protein marker and, most importantly, functional responses of cortical neurons. These characteristics persisted throughout the first week in culture. We believe this protocol can be adopted to isolate and culture the neurons of other ectothermic vertebrate species to further our understanding of anoxia tolerance and other neurophysiological processes.

## Supplementary Material

10.1242/jexbio.250788_sup1 Supplementary information
