# Supplementary material for: A primary neuron culture system for functional studies of anoxia tolerance in turtles
Source: J Exp Biol. 2025 Dec 18;228(24):jeb250788. doi: 10.1242/jeb.250788 (PMC12752498; doi:10.1242/jeb.250788)
Supplement: Supplementary information [file jexbio-228-250788-s1.pdf]

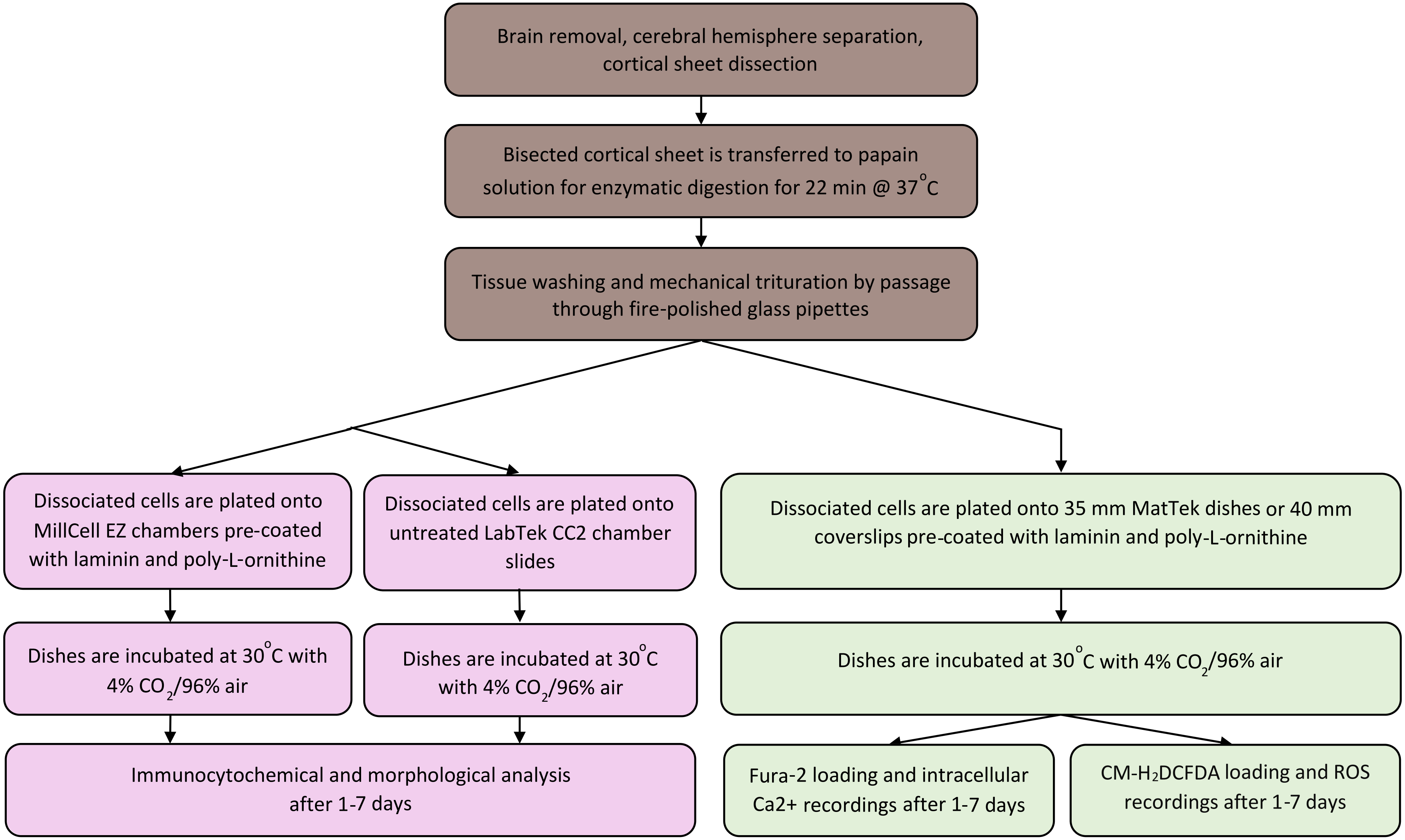

Fig. S1. Workflow chart summarizing the main steps of the turtle neuronal isolation and plating of cells for the different characterization studies. Brain and cortical dissection are color coded in brown, cell plating for morphological and immunolabeling studies are color-coded in pink, and cell plating for functional measurements are color coded in green.

Journal of Experimental Biology • Supplementary information

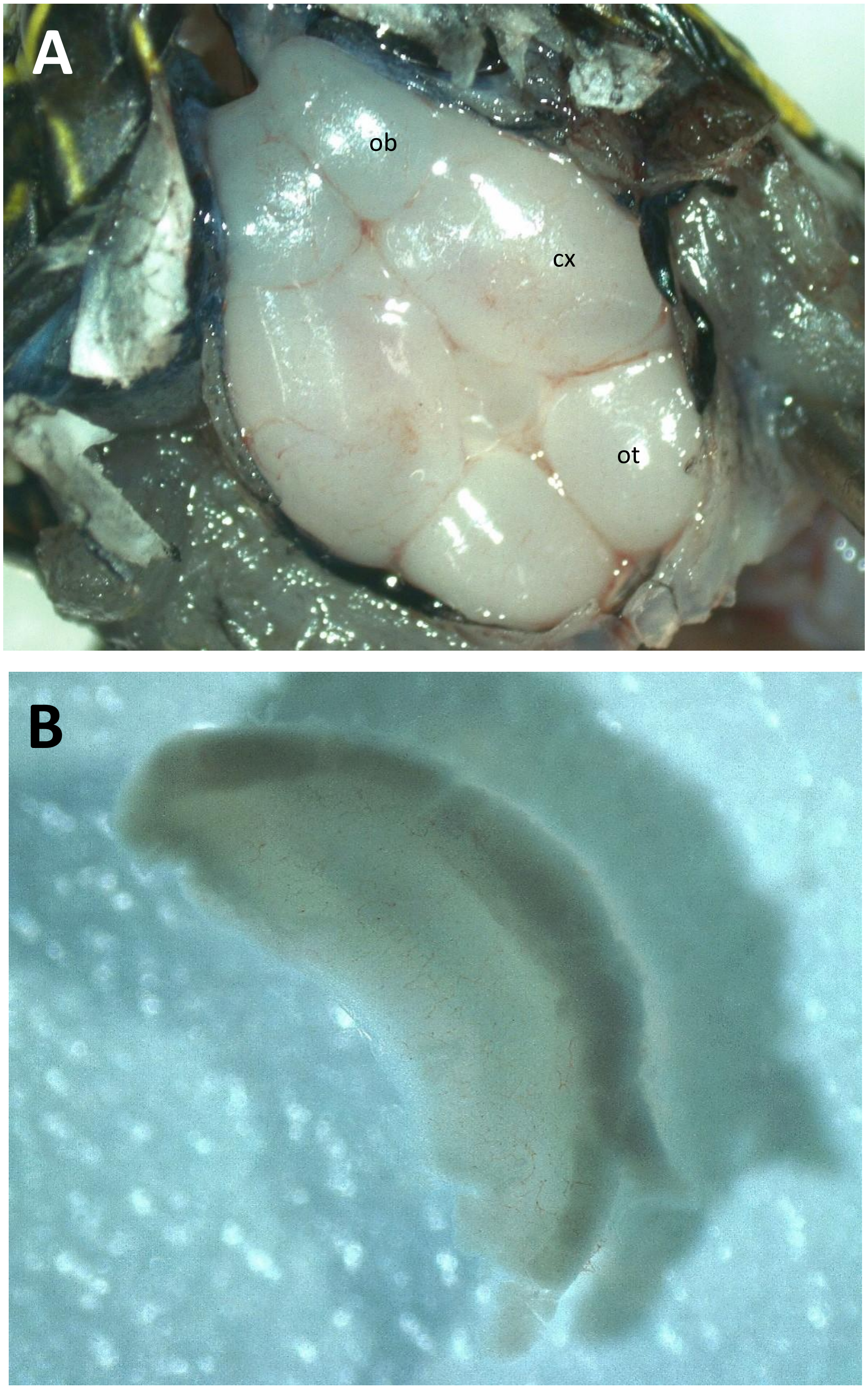

Fig. S2. Photographs of the turtle brain within the braincase (A) prior to extraction and a cortical sheet (B) after dissection from whole brain immediately prior to bisection and transfer to the papain solution. ob=olfactory bulb, cx=cerebrocortex, and ot=optic tectum

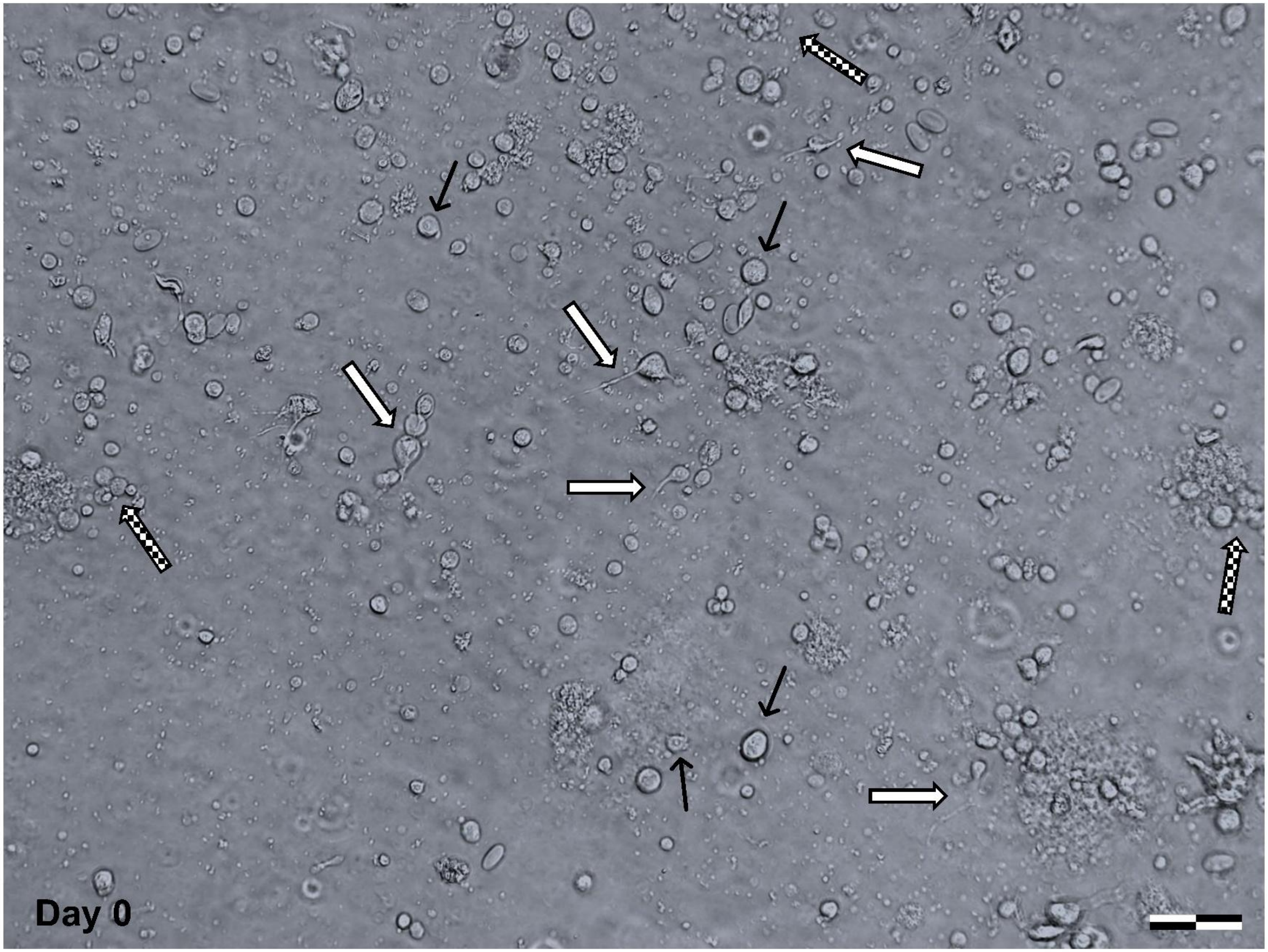

Fig. S3. Examples of isolated primary neurons on day 0 after dissociation from cortex. Dissociates cells were examined right after isolation, before plating in slides and dishes.Numerous cells with round or tear-shaped soma and without neurites were observed, indicated by thin arrows. Several tear-shaped cells with neurites can also be observed, indicated by whitearrows. Clumped cells that were not completely dissociated were observed, indicated by the checkered arrow.Scale bar = 25 µm

Journal of Experimental Biology • Supplementary information

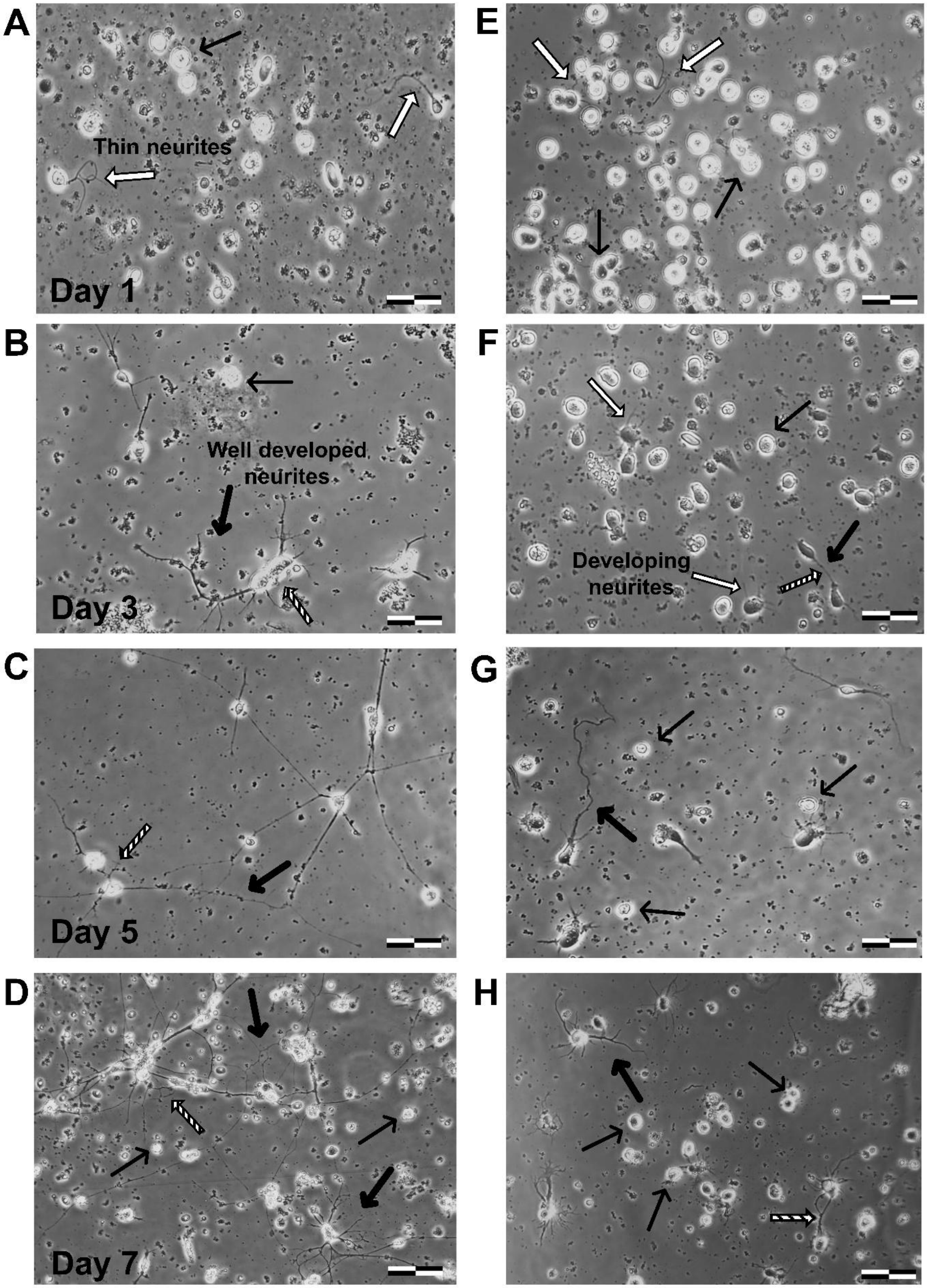

Journal of Experimental Biology • Supplementary information

Fig. S4. Examples of isolated primary neurons cultured for up to seven days in two distinct substrates. Isolated turtle neurons were initially cultured in two substrates – laminin/poly-L substrate because neurons exhibited well-developed neurites earlier in culture. A and E. Isolated primary cortical neurons on day 1 in culture under bright-field conditions. Cells were bright, with round or teardrop shaped soma indicated by thin arrows. Neurites, indicated by the open white arrow, were present in a few cells for both substrates. B and F. On day 3, cells with teardropshaped soma had more developed, thin neurites on L/P (B) when compared to less developed neurites in CC2™ (F). CC2™ also had larger numbers of cells without neurites. Spiny, thin neurites indicated by thick black arrow. Synapses started to be observed. Synapses indicated by striped arrows. C and G. On day 5, cells showed developing basal and apical branched neurites. Longer and more developed neurites, as well as synapses, were more commonly observed on L/P (C) than CC2™ (G). D and H. On day 7, cells showed well developed neurites. Cells cultured in L/P (D) had longer and thinner neurites, synapses were abundant in densely seeded areas, and few cellswere found without neurites. Cells culture in CC2™ had thicker neurites, synapsing cells, and a larger number of cells remained without or with small neurites. Scale bar = 25 µm

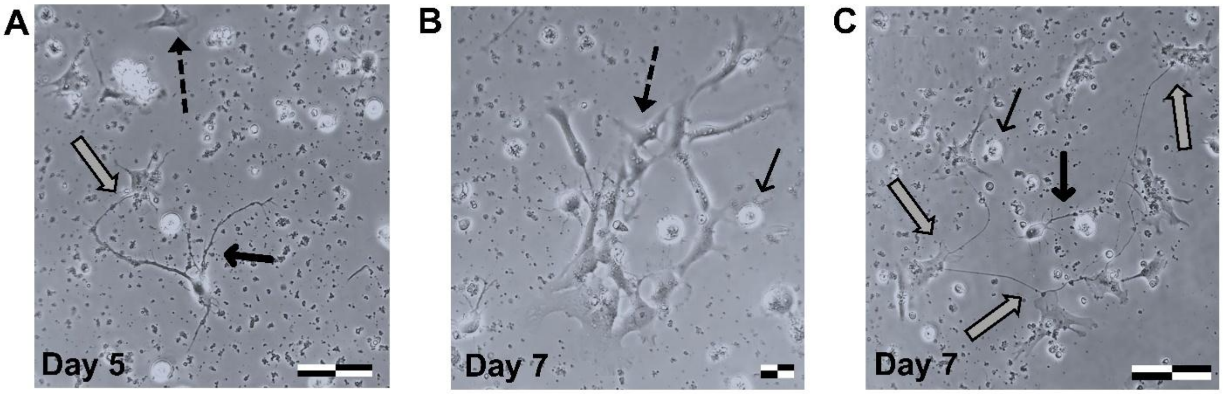

Fig. S5. Examples of fibroblasts on days 5 and 7 in culture. A. Contact point between isolated cortical neuron with well-developed neurites and fibroblast, indicated by gray arrow. Cells cultured on CC2 slide, on day 5 in culture. Scale bar = 21 µm. B. Fibroblasts in confluence on day 7 on an CC2 slide. Cells without neurites and with developing neurites are observed. Scale bar = 13 µm. C. Contact points (gray arrows) between a well-developed neuron and multiple fibroblasts on day 7 in a L/P slide. Scale bar = 21 µm.

Table S1. Morphological measurements of primary cortical cells isolated from young painted turtles (n=3 turtles) and cultured for up to 7 days (DIC).

DIC	Soma Length of Cortical Cells (µm)	# of Cortical Cells	Soma Length of Neurons with Neurites (µm)	Main Neurite Length (µm)	Neurites per Neuron	# of Neurons
1	12.62 ± 0.15	828	13.02 ± 0.37	35.23 ± 2.42	2.47 ± 0.24	94
3	13.84 ± 0.16	829	15.01 ± 0.31	53.65 ± 4.45	2.70 ± 0.15	239
5	11.71 ± 0.18	782	12.34 ± 0.25	74.78 ± 5.09	4.10 ± 0.22	313
7	16.52 ± 0.20	859	18.41 ± 0.26	114.79 ± 5.34	4.72 ± 0.22	430

DIC= days in culture

Table S2. Comparison of poly-l-ornithine/laminin coated to CC2 chamber slides on growth and development of primary cortical cells isolated from young painted turtles (n=2 turtles) and cultured for up to 7 days

DIC	Soma Length of Cortical Cells (µm)		# of Cortical Cells Measured		Soma Length of Neurons with Neurites (µm)		Main Neurite Length (µm)		Neurites per Neuron		# of Neurons	
	PLO/Lam	CC2	PLO/Lam	CC2	PLO/Lam	CC2	PLO/Lam	CC2	PLO/Lam	CC2	PLO/Lam	CC2
1	10.99 ± 0.20	14.45 ± 0.20	365	399	12.63 ± 0.79	13.72 ± 0.47	38.58 ± 4.75	33.06 ± 3.54	2.23 ± 0.35	2.27 ± 0.33	30	47
3	13.87 ± 0.21	14.07 ± 0.23	432	371	16.31 ± 0.53	14.53 ± 0.36	89.93 ± 8.95	27.42 ± 2.17	2.90 ± 0.23	2.58 ± 0.23	98	121
5	9.41 ± 0.16	14.07 ± 0.32	378	351	10.43 ± 0.20	16.19 ± 0.49	74.61 ± 5.28	49.73 ± 6.04	3.70 ± 0.20	5.22 ± 0.60	188	92
7	17.43 ± 0.21	16.50 ± 0.45	610	175	18.97 ± 0.27	19.39 ± 0.75	122.88 ± 6.26	61.65 ± 3.38	4.10 ± 0.18	9.19 ± 1.08	342	56

DIC=days in culture
PLO/Lam is Poly-L-Ornithine/Laminin-coated chamber slides
